# “High Frequency/Small Tidal Volume Differential Lung Ventilation”: A Technique of Ventilating the Nondependent Lung of One Lung Ventilation for Robotically Assisted Thoracic Surgery

**DOI:** 10.1155/2015/631450

**Published:** 2015-08-12

**Authors:** Bassam M. Shoman, Hany O. Ragab, Ammar Mustafa, Rashid Mazhar

**Affiliations:** Cardiothoracic Anesthesia Department, Heart Hospital, Hamad Medical Corporation, P.O. Box 3050, Doha, Qatar

## Abstract

With the introduction of new techniques and advances in the thoracic surgery fields, challenges to the anesthesia techniques had became increasingly exponential. One of the great improvements that took place in the thoracic surgical field was the use of the robotically assisted thoracic surgical procedure and minimally invasive endoscopic thoracic surgery. One lung ventilation technique represents the core anesthetic management for the success of those surgical procedures. Even with the use of effective one lung ventilation, the patient hemodynamics and respiratory parameters could be deranged and could not be tolerating the procedure that could compromise the end result of surgery. We are presenting our experience in managing one patient who suffered persistent hypoxia and hemodynamic instability with one lung ventilation for robotically assisted thymectomy procedure and how it was managed till the completion of the surgery successfully.

## 1. Introduction

The development of lung isolation and one lung ventilation (OLV) accelerated the evolution of thoracic surgery as a subspecialty. Before the introduction of endotracheal tube and the cuffed endotracheal tube, only select few intrathoracic procedures were feasible. Rapid lung movement and quickly developing respiratory distress made the surgical procedures difficult and risky. Selective ventilation of one lung changed this scenario. It was first described in 1931 by Gale and Water and quickly led to increasingly complex lung resection surgery, with the first published pneumonectomy for cancer in 1933 [1]. Techniques and apparatus used for OLV have changed significantly in recent years. These changes have come largely in response to an increased use of OLV during lung surgery and the advent of newer, minimally invasive surgical procedures, whereas OLV in the operating room or intensive care unit was once viewed as a complex endeavor largely managed by experts in academic institutions. The introduction of newer limited access thoracic and cardiac procedures has made it necessary as anesthesia staff members to master lung isolation techniques. Modification of OLV technique is sometimes needed during the procedure to face the potential problems that could change the plans and covert the procedure to conventional lung ventilation. The well-known methods of increasing FIO_2_, applying PEEP to the ventilated lung, use of CPAP to the nonventilated lung, or intermittent reinflation of the collapsed lung may not work to improve hypoxia and hypercarbia associated with OLV.

In this case report we are presenting a modification of the differential lung ventilation technique for managing hypoxia and hypercarbia during robotic assisted thymectomy using OLV [2].

## 2. Case Report

A 35-year-old female Asian patient who is known to be nonsmoker and nonalcoholic referred by the infection control department to the cardiothoracic surgery team after being treated from military tuberculosis by short-term antituberculous regimen for 4 months. She had no neurological signs or symptoms of note. Her physical examination was unremarkable. She was a small-sized person with a body weight of 42 Kg and height of 142 cm.

Routine laboratory works were within normal limits; sputum microscopy was negative for acid-fast bacilli and no mycobacterium was isolated with culture and sensitivity tests. Also serology tests are negative for HbsAg, HCV, and HIV.

CT scan of chest had shown an anterior mediastinal mass, which measured 1.8 cm in its maximum anteroposterior diameter and 4.8 cm in its side-to-side dimensions with multiple peripherally located reticulonodular opacities suggesting tuberculous infection. Chest X-ray showed multiple faint nodular opacities noted in the right apical region with midprominence of the right hilar vascular shadows. The left lung field and both costophrenic angles were clear.

Patient was scheduled for robotic assisted thymus tumor excision with a working diagnosis of Thymic TB versus Thymoma.

On the day of the operation, her vital signs were HR 120/min, sinus rhythm, BP 150/80 mmHg, and SpO_2_ 98% on room air.

In the operation theater, patient was prepared by applying left peripheral venous cannula and right radial artery cannula. Monitoring intraoperatively consists of 5 leads ECG, pulse oximetry, invasive and noninvasive blood pressure, nasopharyngeal temperature, urine output, and bispectral index. Respiratory parameters' monitoring consisted of peak inspiratory pressure, gas analyzer, and O_2_ monitor.

Anesthesia was induced by intravenous injection of propofol 2.5 mg/kg, fentanyl 1.5 mcg/kg, and cisatracurium 0.2 mg/kg followed by endotracheal intubation by double lumen Rusch Robertshaw Endobronchial Tubes Left Bronchus of 35F size and secured at 30 cm at lip level. Position is confirmed by routine breath sound auscultation algorithm. Tube position could not be checked by fiberoptic bronchoscopy only in this instance, as it was not available in theatre on the day of surgery for logistic reason of repair and disinfection. Two anesthetists reconfirm the position by auscultating all chest quadrants and again after port access of the thoracoscope with visual examination of proper lung ventilation during single lung ventilation.

Anesthesia was maintained by intermittent doses of muscle relaxants and fentanyl with inhalational sevoflurane anesthesia.

Patient was placed in supine position with a 30-degree leftward tilt. The endotracheal tube position was reconfirmed after position by auscultating all chest quadrants for effective OLV. The dependent lung was ventilated at respiratory rate of 14/minute, tidal volume of 350 mL, and PEEP of 5 cm H_2_O.

Surgery was performed using a da Vinci Si Robotic system, with four portals. A 7 × 5 cm bulky highly vascular Thymus was excised clearing the region between phrenic to phrenic nerves and vertically from innominate vein till diaphragm.

After starting OLV the patient developed desaturation up to 87% with gradually rising up of ETCO_2_ to 45 mmHg. To solve the problem, FIO_2_ was first increased to 0.1 and then 6 cm H_2_O PEEP applied to the ventilated lung and 5 cm H_2_O CPAP applied to the nonventilated lung but patients' condition unfortunately did not improve. Intermittent reinflation of the collapsed lung by manual ventilation was done to overcome the desaturation problem with marked improvement of SpO_2_ to 100% immediately. Again right lung was collapsed to resume the procedure with trial of pressure-controlled ventilation to the ventilated lung but again this maneuver did not improve the condition. We noticed that ventilating the collapsed lung manually with very small tidal volume and at a high rate of 35 to 40 breath per minute (i.e., pediatric mode of ventilation) through the CPAP circuit did not interfere with the surgical field exposure. In fact, the maneuver maintained SpO_2_ saturation to 99-100% and ETCO_2_ remained below 40 mmHg. ABG done after 10 minutes of this method of ventilation showed PaO_2_ of 217 mmHg and PaCO_2_ of 44 mmHg. Portable ventilation is connected to the nonventilated lung double lumen tube limb with pediatric mode of ventilation of tidal volume of 60 mL, respiratory rate of 35/minute, PEEP of 2 cm H_2_O in order not to interfere with the surgical field, and I : E ratio range from 1 : 2 to 1 : 3. Patient maintained SpO_2_ of 99-100% and ETCO_2_ of 35–38 mmHg all through the procedure. The dependent lung was continued to be ventilated through the anesthesia machine ventilator at the same starting parameters. Surgery was accomplished robotically with complete excision of the tumor without any interfering effect of this mode of ventilation to the collapsed right lung.

At the end of the procedure, patient was extubated on the operating table and transferred to the recovery room for 2 hours monitoring and then transferred to wardroom. Patient was discharged from the hospital on the 5th postoperative day and followed up in the thoracic surgery clinic with normal postprocedure course.

## 3. Discussion

Differential lung ventilation is a well-known technique for ventilating patient with unilateral lung disease in the critical care settings in the ICU [3]. However, using this technique intraoperatively in the operative suite setting is rarely applied. Conventional one lung ventilation technique provides satisfactory gas exchange in the majority of cases. However, in some cases hypoxemia may occur secondary to the obligatory right to left transpulmonary shunt through the nonventilated, nondependent lung [4]. Another factor which could be added to the presented case is the past history of miliary pulmonary tuberculosis 6 months prior to the procedure. These factors will result in a much larger alveolar arterial oxygen difference and lower PaO_2_ than does OLV. However, blood flow to the nonventilated lung is usually reduced by gravity in the complete lateral decubitus position, active hypoxic pulmonary vasoconstriction in the nonventilated lung, and nondependent lung collapse [5]. Watanabe et al. had investigated the effect of gravity as a major determinate of shunt and perfusion during thoracotomy procedures. Patients undergoing right thoracotomy were divided into three groups. One group was supine, one group was placed in the left semilateral decubitus position, and the third group was placed in the left full-lateral position. All patients were ventilated with 100% oxygen, and arterial blood gas samples were analyzed every 5 min after intentional collapse of the right lung. PaO_2_ progressively decreased in all groups after two-lung ventilation (TLV) was discontinued. Nine out of 11 patients in the supine group experienced arterial oxyhemoglobin saturation (SaO_2_) of less than 90% and had to have TLV reinstituted. Only one out of nine patients in the semilateral group and one out of 13 patients in the full-lateral group experienced that degree of hypoxemia. The time for PaO_2_ to decrease to 200 mmHg after the start of SLV was very rapid: 354 s in the supine group compared with 583 s in the semilateral group and 794 s in the full-lateral group [6].

Bardoczky et al. compared the effects of position and fraction of inspired oxygen (FiO_2_) during thoracic surgery. Randomly assigned patients were ventilated with a FiO_2_ of 0.4, 0.6, or 1.0 during periods of TLV and SLV in the supine and lateral positions. PaO_2_ decreased more during SLV compared with TLV in all groups in both positions. In all three groups PaO_2_ was significantly higher during SLV in the lateral than in the supine position [7]. Those studies demonstrated that, during SLV with a patient in the full-lateral position, gravity augments the redistribution of perfusion to the ventilated lung, resulting in a better V/Q match and a higher PaO_2_.

As a result of these factors, the degree of shunt and hence conventional ventilation with 100% oxygen is usually associated with accepted PaO_2_ values. As in robotic thoracic surgery, partial lateral decubitus position with 30–45 degree surgery side up is utilized; blood flow to the nondependent lung is not completely reduced to decrease the shunt fraction, attributing to patient hypoxia as another added factor. As we had CPAP breathing circuit applied to the nondependent limb of the double lumen tube, the main advantage of this breathing circuit is that it has small reservoir bag that can be utilized for manually ventilating the collapsed lung with small pediatric tidal volumes simulating baby lung ventilation. Manually ventilating the lung all throughout the whole length of the procedure is not practically feasible in all situations and subjected to personal variations. Mechanically ventilating the nondependent lung with portable ventilator utilizing the pediatric mode of ventilation can replace this manual technique. We find it very effective in improving the oxygenation of the patient with decreased FIO_2_ of the ventilated lung to 0.8 and at the same time, not disturbing the operative field.

Differential lung ventilation can be used during thoracotomy, VATS procedure, or robotically assisted thoracoscopic cardiac or thoracic surgery whenever OLV is followed by hypoxemia despite adequate ventilation of the dependent lung with 100% oxygen and failed other techniques to maintain oxygen saturation [8].
